# Pratiques contraceptives des femmes infectées par le VIH suivies en ambulatoire au Centre Hospitalier Universitaire de Treichville (Abidjan, Côte d'Ivoire)

**DOI:** 10.11604/pamj.2019.33.79.16435

**Published:** 2019-06-03

**Authors:** Edouard N'guessan, Franck Gbeli, Jean Marc Dia, Privat Guie

**Affiliations:** 1 Service de Gynéco-obstétrique, CHU de Treichville, Abidjan, Côte d'Ivoire

**Keywords:** Planification familiale, contraception, infection à VIH, Family planning, contraception, HIV infection

## Abstract

**Introduction:**

La planification familiale est une stratégie à haut impacts pour la réduction de la mortalité maternelle et pour la prévention de la transmission mère-enfant du VIH. L'objectif de cette étude était de décrire les pratiques contraceptives des femmes infectées par le VIH suivies en ambulatoire au CHU de Treichville

**Méthodes:**

Une enquête transversale descriptive a été réalisée dans les unités de soins ambulatoires aux personnes vivant avec le VIH du CHU de Treichville du 1^er^ avril au 30 juin 2016. Durant cette période, toutes les patientes VIH positives en âge de procréer, fréquentant les services de gynécologie-obstétrique, pneumo-phtisiologie, dermatovénérologie et médecine interne ont été invitées à renseigner un questionnaire standardisé portant sur les caractéristiques sociodémographiques, médicaux et les pratiques contraceptives

**Résultats:**

Au total, 283 femmes ont accepté de participer à l'étude, l'âge médian était de 36 ans avec une parité moyenne de 1,7. Les patientes étaient nullipares dans seulement 22,3% des cas et vivaient en couple dans 54,8% des cas. Elles n'avaient pas d'enfant avec le conjoint actuel dans 68,2% des cas. Le conjoint était informé du statut VIH dans 51,6% des cas. Elles étaient sous traitement antirétroviral dans 92,9% des cas avec une médiane de CD4 de 382 éléments/ml. La majorité des patientes (62,9%) avaient déclaré utiliser une méthode contraceptive moderne. Elles utilisaient surtout les progestatifs injectables (45,5%) et l'implant (32,6%). La pratique de la double protection n'a été signalé par seulement 17,4% d'entre-elles. Le niveau scolaire secondaire et supérieur (OR=2,23 [1,35-3,69], p=0,01), la multiparité (OR=1,84 [1,11-3,06], p=,002) et la révélation du statut VIH au conjoint (OR=1,86 [1,14-3,03], p < 0,01) étaient les facteurs significativement associés à l'utilisation de la contraception

**Conclusion:**

Les pratiques contraceptives des femmes infectées par le VIH dans notre expérience restent globalement décevantes. Il faut développer des stratégies visant à améliorer l'intégration de la planification familiale dans la prise en charge les femmes infectées par le VIH.

## Abstract (en)

**Introduction:**

Family planning is a high impact strategy to reduce maternal mortality and to prevent mother-to-child transmission of HIV. This study aims to describe contraceptive practices of HIV-infected women followed upon an ambulatory basis at the Treichville University Hospital.

**Methods:**

We conducted a cross-sectional descriptive multicenter survey of people living with HIV in the ambulatory care units of the Treichville University Hospital from 1 April to 30 June 2016. During this period, all HIV positive patients of childbearing age attending the Department of Obstetrics and Gynecology, the Pneumophtisiology Department, the Department of Dermatovenereology and the Department of internal medicine were invited to complete a standardized questionnaire on the sociodemographic, medical characteristics and the contraceptive practices

**Results:**

In total, 283 women agreed to participate in the study, their median age was 36 years with an average parity of 1.7. Patients were nulliparous in only 22.3% of cases and lived with a partner in 54.8% of cases. They had no child with the current partner in 68.2% of cases. The partner was informed about their HIV status in 51.6% of cases. They were under antiretroviral treatment in 92.9% of cases with a median mean CD4 of 382 cells/mL. The majority of patients (62.9%) declared that they were using a modern contraceptive method. They mainly used progestin injectable (45.5%) and contraceptive implant (32.6%). The practice of dual protection was reported by only 17.4% of them. Secondary and higher education (OR=2.23 [1,35-3,69], p=0.01), multiparity (OR=1.84 [1,11-3,06], p=0.002) and revelation of HIV positive status to the partner (OR=1.86 [1,14-3,03], p<0.01) were factors significantly associated with the use of contraception

**Conclusion:**

Based on our experience, contraceptive practices in women infected with HIV are generally discouraging. It is essential to develop strategies to improve the integration of family planning into the management of HIV-infected women.

## Introduction

Les femmes en âge de procréer en Afrique sub-saharienne supportent à la fois le fardeau de l'infection par le Virus de l'Immunodéficience Humaine (VIH) et celui de la mortalité maternelle. En effet, d'après le Programme de l'Organisation des Nations Unies sur le SIDA (ONUSIDA) près de 60% des adultes infectées par le VIH dans cette région sont des femmes en âge de procréer [1]. De plus, cette région concentre à elle seule, plus de la moitié des décès maternels enregistrés chaque année à travers le monde [2]. Fournir l'accès à la planification familiale aux femmes afin de prévenir les grossesses non planifiées et non désirées est une intervention à hauts impacts sur la réduction de la mortalité maternelle [3]. Chez les femmes infectées par le VIH, la planification familiale a été identifiée comme une stratégie majeure qui permet de réduire à la fois la mortalité maternelle et la transmission mère-enfant de l'infection à VIH [4]. C'est pourquoi, la prévention des grossesses non désirées chez les femmes vivant avec le VIH a été retenue par l'Organisation Mondiale de la Santé (OMS) comme l'un des quatre piliers de sa stratégie globale pour l'élimination de la transmission mère-enfant de l'infection à VIH [5]. L'atteinte de cet objectif clé exige d'augmenter l'accès et l'utilisation des méthodes contraceptives modernes par les femmes infectées par le VIH. Or, des études ont montré que presque tous les pays d'Afrique sub-saharienne ont des prévalences faibles d'utilisation de contraceptifs et des besoins non satisfaits en contraception élevés [6]. En outre, des enquêtes menées auprès des femmes infectées par le VIH en Afrique [7] et aux USA [8] ont montré des taux élevés de grossesses non désirées chez ces dernières. La Côte d'Ivoire, avec une séroprévalence de 2,7% et environ 500 000 personnes vivant avec le VIH (PVVIH) est l'un des pays les plus touchés par l'infection à VIH/SIDA en Afrique de l'Ouest [1]. Dans ce pays, 59,5% des personnes de 15-49 ans infectées sont des femmes. Par ailleurs, notre pays enregistre un indice synthétique de fécondité élevé à 5 enfants par femme avec une prévalence de l'utilisation de la contraception faible à 14% et des besoins non satisfait en contraception élevés à 27% [9]. Toutefois, ces indicateurs de santé reproductive concernent la population générale et il existe peu de données sur le cas spécifique des femmes infectées par le VIH. L'objectif de ce travail était de décrire les pratiques contraceptives des femmes infectées par le VIH suivies en ambulatoire au CHU de Treichville.

## Méthodes

**Types d'étude:** ce travail est une enquête transversale réalisée dans les unités de soins ambulatoires aux personnes adultes vivant avec le VIH de quatre services du CHU de Treichville (Dermatologie-Vénérologie, Gynécologie-Obstétrique, Pneumo-Phtisiologie, et Médecine interne). Chacun de ces services dispose depuis décembre 2006 d'une unité de suivi médical des personnes vivant avec le VIH et ceci dans le cadre de la politique de renforcement et de décentralisation de la prise en charge des PVVIH. La période d'inclusion a duré trois mois (allant du 1^er^ avril au 30 juin 2016).

**Population d'étude:** durant la période de l'étude, toutes les femmes infectées par le VIH, âgée de 15 à 45 ans qui avaient consulté en ambulatoire dans l'un de ces quatre services ont été invitées à participer à l'étude. Les critères de non inclusion étaient: les femmes non consentantes à participer à l'étude, les femmes enceintes et celles qui avaient une infertilité définitive.

**Collecte et analyse des données:** les données ont été collectées après consentement éclairé, à l'aide d'un questionnaire standardisée lors d'un entretien individuel mené par des enquêteurs formés. Ces enquêteurs étaient tous des conseillères communautaires travaillant dans ces différentes unités de suivi des PVVIH. Les variables étudiées ont porté sur les données sociodémographiques, les données liées à l'infection à VIH et les données liées à la pratique contraceptive. Les caractéristiques sociodémographiques concernaient: l'âge, la profession, le niveau d'instruction scolaire, le statut matrimonial, la parité, le nombre d'enfant vivant et le nombre d'enfant avec le conjoint actuel. Tandis que les données liées à l'infection à VIH étaient: la durée de la séropositivité, le traitement antirétroviral, le taux de CD4 et la divulgation du statut au conjoint. Quant aux données liées à la pratique contraceptive, elles portaient sur: l'utilisation d'un contraceptif moderne c'est-à-dire hormonal ou mécanique, le type de contraceptif, la pratique de la double protection. Les contraceptifs considérés comme modernes dans cette études étaient: les contraceptifs hormonaux (progestatifs injectables, pilules estroprogestatives, micropilules progestatives pures et implant progestatif sous-cutané) et les contraceptifs mécaniques (préservatif masculin et dispositif intra-utérin au cuivre). La pratique de la double protection consiste en l'utilisation simultanée du préservatif associé à une autre méthode contraceptive hormonale ou mécanique. L'analyse des données a été effectuée à l'aide du logiciel SPSS.22. Les facteurs associés à l'utilisation de la contraception par les femmes vivant avec le VIH ont été recherchée à l'aide du Odds ratio avec un intervalle de confiance à 95% et du test du chi2. Une valeur p < 0,05 a été retenue comme seuil de signification.

**Considérations éthiques:** le comité d'éthique institutionnel de la direction médicale et scientifique du CHU de Treicheiville a donné son approbation pour la réalisation de l'étude et toutes les participantes ont donné leur consentement éclairé avant leur enrôlement dans l'étude. Les entretiens ont été réalisés dans les conditions optimales d'écoute et de confidentialité.

## Résultats

**Figure 1:**
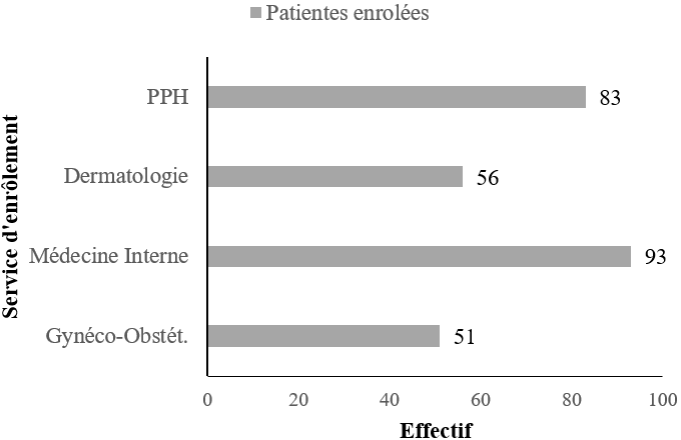
Diagramme expliquant le processus d'enrôlement dans l'étude

**Description de la population d'étude:** sur un total de 310 patientes infectées par le VIH approchées, 286 ont accepté de participer à l'étude. Parmi elles, 3 avaient infertilité définitive. En définitive, l'analyse a concerné 283 patientes soit un taux de réponse de 91,3% (Figure 1). Les caractéristiques sociodémographiques et médicales des femmes infectées par le VIH ayant participé à l'étude sont présentées dans le Tableau 1. Elles avaient un âge médian de 36 ans (écart interquartile: 31-37 ans) avec une parité médiane à 2 (extrêmes: 0 à 9). Parmi elles, 155 (54,8%) vivaient en couple. Elles étaient nullipares dans 22,3% des cas et n'avaient d'enfant vivant dans 30,7% des cas. La majorité d'entre-elles (68,2%) n'avait pas d'enfant avec le conjoint actuel. Alors que 93,6% des femmes était sous trithérapie antirétrovirale, seulement 51,6% d'entre-elles avait révélé leur statut VIH à leur conjoint.

**Tableau 1:** Caractéristiques de la population d’étude

Caractéristiques	Effectifs (n)	Pourcentage (%)
**Age maternel (ans)**		
20-29	44	15,5
30-39	152	53,7
40-45	87	30,8
**Profession**		
Ménagère	113	40,0
Libérale	111	69,2
Salariée	53	18,7
Elève/étudiante	6	2,1
**Niveau d’éducation scolaire**		
Non scolarisé	56	19,8
Primaire	98	34,6
Secondaire	97	34,3
Supérieur	32	11,3
**Statut matrimonial**		
Femme seule	128	45,2
En couple	155	54,8
**Parité**		
0	64	2,6
1-2	30	10,6
> 2	189	66,8
**Nombre d’enfants vivants**		
0	87	30,7
1-2	87	30,7
> 2	109	38,5
**Nombre d’enfants avec conjoint**		
0	193	68,2
1-2	39	13,8
> 2	51	1,0
Désir d’avoir des enfants plus tard		
Oui	212	74,9
Non	71	25,1
**Traitement antiretroviral**		
Oui	265	93,6
Non	18	6,4
**Taux de CD4 (cellules/ml)**		
<350	113	40,0
350-499	82	28,9
≥ 500	88	31,1
**Révélation du statut VIH au conjoint**		
Oui	146	51,6
Non	137	48,4

**Prévalence et pratique contraceptive:** parmi les 283 femmes infectées par le VIH dans cette série, 178 avaient déclaré utiliser une méthode contraceptive moderne soit une prévalence de l'utilisation de la contraception de 62,9%avec un intervalle de confiance (IC) à 95% compris entre 57,4-68,6%. Les pratiques contraceptives des 178 enquêtées ayant déclaré utiliser une méthode contraceptive moderne sont résumées dans le Tableau 2. Les progestatifs injectables avec un taux de 45,5% étaient la méthode la plus utilisée. Seulement 17,4% d'entre-elles pratiquait la double protection.

**Tableau 2:** Pratique contraceptive des femmes infectées par le VIH (n=178)

Pratique contraceptive	Effectifs (n)	Pourcentage (%)
**Type de contraceptif**		
Progestatif injectable	81	45,5
Implant sous-cutané	58	32,6
Contraceptif oral	22	12,4
Dispositif intra-utérin (DIU)	17	9,5
**Double protection**		
Oui	31	17,4
Non	147	82,6

**Facteurs associés à l'utilisation de la contraception:** les résultats de l'analyse statistique sont présentés dans le Tableau 3. L'utilisation de la contraception par les femmes vivant avec le VIH était significativement associée au bon niveau scolaire (OR=2,23 [1,35-3,69], p=0,01), à la multiparité (OR=1,84 [1,11-3,06], p=,002) et à la révélation du statut VIH au conjoint (OR=1,86 [1,14-3,03], p < 0,01) (Tableau 3).

**Tableau 3:** Facteurs associés à l’utilisation de la contraception

	Utilisation de contraception		P-value	Odds ratio (IC)
	Oui (n=178)	Non (n=105)		
	n(%)	n(%)		
**Age maternel (ans)**				
≥ 30	147(82,6)	92(87,6)		
< 30	31(17,4)	13(17,4)	0,26	0,67 [0,33-1,35]
**Profession**				
Sans revenu	80(44,9)	39(37,1)		
Avec revenu	98(55,1)	66(67,9)	0,20	1,38 [0,84-2,26]
**Niveau scolaire**				
Secondaire et supérieur	94(52,8)	35(33,3)		
Aucun et primaire	84(47,2)	70(66,7)	**0,01**	**2,23 [1,35-3,69]**
**Statut matrimonial**				
Femme seule	80(44,9)	48(45,7)		
En couple	98(55,1)	57(54,3)	0,89	0,97 [0,59-1,57]
**Parité**				
> 2	128(71,9)	61(58,1)		
≤ 2	50(28,1)	44(46,9)	**0,02**	**1,84 [1,11-3,06]**
**Nombre d’enfants vivants**				
> 2	66(37,1)	43(40,9)		
≤ 2	112(62,9)	62(59,1)	0,41	0,85 [0,52-1,39]
**Enfants avec conjoint**				
Oui	31(17,4)	20(10,1)		
Non	147(82,6)	85(80,9)	0,73	0,89 [0,48-1,67]
**Désir d’enfants**				
Non	135(75,8)	77(73,3)		
Oui	43(24,2)	28(26,7)	0,64	1,41 [0,65-1,98]
**Traitement antirétroviral**				
Oui	168(94,4)	97(92,4)		
Non	10(5,6)	8(7,6)	0,50	1,38 [0,53-3,32]
**Taux de CD4 (cellules/ml)**				
≥ 350	102(57,3)	68(64,8)		
> 350	76(42,7)	37(35,2)	0,21	1,73 [0,44-1,20]
**Statut VIH révélé au conjoint**				
Oui	102(57,3)	44(41,9)		
Non	76(42,7)	61(58,1)	**0,01**	**1,86 [1,14-3,03]**

## Discussion

### Prévalence contraceptive

Dans la présente étude, la prévalence contraceptive chez les femmes infectées par le VIH était de 62,5%. Plusieurs études en Afrique sub-saharienne ont évalué l'utilisation des méthodes contraceptives modernes chez les femmes infectées par le VIH. La proportion de femmes infectées par le VIH utilisant des contraceptifs dans cette étude était similaire à celle observée dans une étude sud-africaine. Dans cette enquête prospective ayant inclus 290 femmes vivant avec le VIH à Durban, la prévalence contraceptive était de 63% [10]. En revanche, des prévalences contraceptives plus faibles que la nôtre ont été rapportées par d'autres auteurs. Ainsi, Samba *et al.* au Ghana [11] et Wekesa *et al.* au Kenya [12] ont retrouvé dans leurs différents travaux des prévalences contraceptives respectivement de 24,7% et 55,5%. Ces différences peuvent s'expliquer par l'inhomogénéité au plan socio-économique des populations d'étude dans ces différents travaux. En effet, il existe dans la littérature sub-saharienne, plusieurs études qui ont montré le rôle déterminant des facteurs socio-économiques dans l'utilisation des méthodes contraceptives modernes chez les femmes vivant avec le VIH [13-15]. Par ailleurs, nous notons aussi que le taux d'utilisation des contraceptifs par les femmes infectées par le VIH retrouvé dans cette série était 4,5 fois plus élevé que la prévalence contraceptive générale au niveau national [9]. Ces mêmes constats ont été faits dans des études menées en Afrique du Sud [10] et en Ethiopie [16]. Ces résultats montrent que l'intégration des services de planification familiale dans les programmes de prise en charge médicale des personnes vivant avec le VIH est associée à une augmentation de l'utilisation des méthodes contraceptives modernes chez les femmes infectées par le VIH [17, 18]. Cette offre de services de planification familiale doit intervenir de façon précoce dès le dépistage et se maintenir tout au long du suivi des femmes vivant avec le VIH en âge de procréer.

### Pratique contraceptive

Dans la série que nous présentons, les progestatifs injectables étaient la méthode contraceptive la plus utilisée par les femmes infectées par le VIH. Des résultats similaires ont été retrouvés dans des études antérieures. En effet, les progestatifs injectables étaient aussi la méthode contraceptive la plus utilisée dans différents travaux menés auprès de femmes vivant avec le VIH au Swaziland [7], en Ethiopie [16] et au Malawi [19]. Nous avons également constaté que l'implant était la deuxième méthode contraceptive la plus utilisée (32,6%), loin devant les contraceptifs oraux (12,4%) et le dispositif intra-utérin (9,5%). Ces résultats montrent la préférence pour l'implant comparé au dispositif intra-utérin des femmes qui optent une méthode contraceptive à longue durée d'action (MCLDA). L'étude de de Sarnquist *et al.* nous conforte dans cette affirmation [4]. Cependant, comparée à la plupart des données publiées dans la littérature d'Afrique sub-saharienne, la proportion des femmes de notre échantillon qui utilisait une MCLDA parait très importante. Dans l'étude de Mayhew *et al.* au Kenya, seulement 16% des 240 femmes vivant avec le VIH interrogées utilisait une MCLDA [20]. Dans celle deLuster *et al.* qui avait porté sur 893 femmes vivant avec le VIH au Malawi, le taux d'utilisation des MCLDA n'était que de 1,4% [21]. Les études ont démontré que le taux d'utilisation des MCLDA par les femmes vivant avec le VIH augmentait de façon substantielle avec l'amélioration des connaissances des prestataires de santé sur ces méthodes, le renforcement de leur compétence en conseil et l'amélioration de la disponibilité de ces méthodes dans les centre de santé [7, 22, 23]. Ces MCLDA et les contraceptifs hormonaux sont efficaces pour prévenir les grossesses non désirées mais ne protègent pas contre la transmission du VIH ou des autres infections sexuellement transmissibles [24]. C'est pourquoi la double protection combinant l'utilisation systématique du préservatif avec une méthode contraceptive efficace est fortement recommandée chez les femmes infectées par le VIH. Cependant, les études indiquent que la pratique de cette double protection en Afrique sub-saharienne est faible et reste généralement inférieur à 20% [25]. Nos résultats sont en accord avec ces observations. Des études expliquent ce faible taux de pratique de la double protection en Afrique sub-saharienne par le caractère stigmatisant de l'utilisation du préservatif dans le mariage dans le contexte culturel africain [26, 27].

### Facteurs associés à l'utilisation de la contraception

L'un des facteurs associés à l'utilisation de la contraception par les femmes vivant avec le VIH était le bon niveau d'éducation scolaire. Le niveau scolaire a été également retrouvé comme facteur prédictif d'utilisation de méthodes contraceptives moderne dans autres études [19]. Ce résultat montre bien que l'éducation est l'un des meilleurs leviers pour améliorer les performances des programmes de santé reproductive. Notre série avait aussi noté une association significative entre l'utilisation de la contraception par les femmes vivant avec le VIH et la multiparité. Notre observation est en accord avec les données de la littérature. En effet, des études antérieures réalisées chez des femmes vivant avec le VIH avaient également montré une association entre le nombre plus élevé d'enfants et l'utilisation de contraceptifs [13, 19]. En outre, on a noté un niveau d'utilisation des méthodes contraceptives significativement plus élevé parmi les femmes ayant révélé leur statut VIH à leur conjoint. Ces résultats montrent que la divulgation du statut VIH au partenaire sexuel, lorsqu'elle est bien conduite permet une meilleure adhésion aux interventions de préventions [28].

**Limites de l'étude:** la présente étude présente quelques limites. En effet, seulement un cinquième des femmes vivant avec le VIH en âge de procréer qui étaient dans la file active durant la période de l'étude ont été concerné par l'enquête. De plus, l'enquête a eu lieu dans un hôpital universitaire qui réunit théoriquement de meilleures conditions de prise en charge pour ces femmes. Par conséquent, la population d'étude peut ne pas être représentative de la population générale des femmes infectées par le VIH pouvant avoir des besoins en planification familiale. On peut donc craindre l'existence d'un biais de sélection qui rendra difficile l'extrapolation de nos résultats à la population générale. En outre, le recueil déclaratif des données est une source potentielle de biais d'information surtout par mensonge.

## Conclusion

Dans cette étude, moins des deux tiers (62,9%) des femmes infectées par le VIH interrogées utilisaient une méthode contraceptive moderne. Cette étude suggère aussi une bonne acceptation de l'implant par les femmes vivant avec le VIH. Toutefois, elle a montré que la pratique de la double protection restait encore faible. Ces résultats montrent que des efforts doivent être encore faits pour augmenter l'accès et l'utilisation des services de planification familiale par les femmes infectées par le VIH dans une zone de forte prévalence du VIH comme la nôtre. Des stratégies visant à assurer l'intégration effective des services de planification familiale aux soins des personnes vivant avec le VIH doivent être développées. Il faut commencer par la formation des professionnels de la santé assurant la prise en charge des personnes vivant avec le VIH en conseils pour la planification familiale et sur les différentes méthodes contraceptives en insistant sur les MCLDA et la double protection. Il faut également permettre au système de santé d'assurer la disponible continue des différentes gammes de contraceptifs.

### Etat des connaissances actuelles sur le sujet

- La mortalité maternelle et l'infection à VIH/SIDA sont deux fléaux qui touchent durement les femmes en âge de procréer en Afrique sub-saharienne;
- Chez les femmes infectées par le VIH, la planification familiale a un double enjeu: réduire la mortalité maternelle et prévenir la transmission mère-enfant du VIH;
- La double protection est la pratique contraceptive recommandée chez les femmes infectées par le VIH;

### Contribution de notre étude à la connaissance

- La prévalence contraceptive chez les femmes infectées par le VIH bien que nettement plus élevée que la prévalence contraceptive nationale reste encore faible;
- On note une très faible utilisation des méthodes contraceptives à longue durée d'action par les femmes infectées par le VIH;
- Le taux de pratique de la double protection est également faible.

## Conflits d’intérêts

Les auteurs ne déclarent aucun conflit d'intérêts.
